# Identification of Prognostic Markers in Argentinian Acute Lymphoblastic Leukemia Pediatric Patients Through Transcriptome Analysis: Differential Gene Expression, Mutational Status and Immune Microenvironment

**DOI:** 10.1200/GO.22.23000

**Published:** 2022-05-05

**Authors:** Mercedes Abbate, María Sol Ruiz, Daniel Avendaño, María Cecilia Riccheri, Elba Vazquez, Javier Cotignola

**Affiliations:** ^1^ CONICET-IQUIBICEN; Dep. Química Biológica-FCEyN-Universidad de Buenos Aires, Buenos Aires, Argentina; ^2^ Hospital Nacional Posadas, El Palomar, Buenos Aires, Argentina

## Abstract

Acute lymphoblastic leukemia (ALL) is the most incident pediatric cancer. Although considerable progress has been made on treatment efficacy and survival rates, 15%-30% of patients relapse and/or die. We aimed to identify molecular profiles and microenvironment makeup in leukemic bone marrow samples that could help to better predict disease outcome.

**METHODS:**

We performed RNA-seq on bone marrow samples from pediatric ALL patients at diagnosis (n = 37). Patients were recruited under a multi-center clinical protocol in Argentina (median follow-up: 31 months). We analyzed differential gene expression, gene set variations, mutations in candidate genes, and fusion genes among clinico-pathological features. The abundance of immune-cell populations was inferred by digital cytometry (MIXTURE) and a “cytolytic score” based on the expression of five genes specific to cytotoxic cells.

**RESULTS:**

We detected: (1) 37 differentially expressed genes (DEG) between poor vs good responders to prednisone; (2) 71 DEG between high vs standard-risk groups; (3) 13 DEG between patients who died/relapsed vs those who did not; and (4) 35 DEG between patients that developed severe therapy-related acute toxicity vs those who did not (|log2FC|> 1; *P*.adj < .05). We observed that 15%-30% of the DEG corresponded to lncRNAs. We found six differentially expressed pathways relevant to cancer and leukocyte biology among risk groups (*P* < .01). We identified 17 mutations and three fusion genes in 44% of patients; the presence of these mutations shortened the relapse-free survival (Cox-*P*-val = .006). High cytolytic score was associated with activated CD8+T cells, immune cell trafficking, and bone marrow niche signaling genesets, suggesting potential candidates for immunotherapies. Higher CD8+T-cell/NK at diagnosis was marginally associated with worse event-free survival (hazard-ratio = 5.39; Cox-*P*-val = .08).

**CONCLUSION:**

Transcriptomic sequencing allowed the analysis and integration of multiple molecular features that might improve pediatric ALL outcome prediction.

